# Digital Quality Monitoring for Type 2 Diabetes in Swiss Primary Care: Qualitative Interview Study

**DOI:** 10.2196/82960

**Published:** 2026-05-12

**Authors:** Odile-Florence Giger, Lola Jo Ackermann, Marinja Principe, Simon Meier, Elgar Fleisch, Susanna Gallani, Michael Brändle, Tobias Kowatsch, Mia Jovanova

**Affiliations:** 1 Centre for Digital Health Interventions, Institute of Technology Management, University of St. Gallen, St. Gallen, Switzerland; 2 Centre for Digital Health Interventions, School of Medicine, University of St. Gallen, Dufourstrasse 40a, St. Gallen, 9000, Switzerland, 41 766300096; 3 Institute for Implementation Science in Health Care, Centre for Digital Health Interventions, University of Zurich, Zurich, Switzerland; 4 Centre for Digital Health Interventions, Department of Management, Technology and Economic, ETH Zurich, Zurich, Switzerland; 5 Accounting and Management Unit, Harvard Business School, Harvard University, Boston, MA, United States; 6 HOCH Health Ostschweiz, Cantonal Hospital St. Gallen, St. Gallen, Switzerland

**Keywords:** type 2 diabetes, patient-centered care, primary care, interviews, Switzerland, integrated care, digital health, qualitative

## Abstract

**Background:**

General practitioners (GPs) manage most type 2 diabetes (T2D) cases worldwide but face increasing workloads. To support guideline-based T2D care, the Swiss Society of Endocrinology and Diabetology (SSED) developed a quality monitoring tool for GPs to track and document patient clinical outcomes, such as glycated hemoglobin testing, blood pressure measurement, and diabetes-related complications in primary care. By aggregating these elements, the SSED score provides a structured way to assess adherence to recommended standards of diabetes care. Although this approach shows promise for improving health outcomes, the score is currently available in paper format and creates an additional administrative burden for physicians.

**Objective:**

This study examined challenges in T2D management in Swiss primary care, assessed limitations of the current SSED quality score, and identified strategies to enable the potential digital implementation and wider adoption of a digital SSED quality score in routine practice.

**Methods:**

A total of 38 qualitative interviews were conducted with 39 participants, representing 4 stakeholder groups relevant to digital T2D care: health care professionals (n=12), individuals with T2D (n=12), software developers (n=10), and health insurer representatives (n=5). Data were analyzed using thematic analysis.

**Results:**

Participants highlighted persistent challenges in T2D management, including time pressure for health care professionals, fragmented care, and a lack of personalized support for individuals with T2D. Barriers to digital quality monitoring included poor integration with GP systems, misaligned incentives, and limited relevance to multimorbidity. Suggested facilitators included embedding digital tools, such as a digital SSED score, into existing workflows, optimizing task shifting for GPs, integrating patient-centered features, and improving data sharing.

**Conclusions:**

A digital SSED score could support GPs by integrating patient-entered data, generating automated alerts, and enabling pharmacy-based follow-up. Successful adoption is likely to depend on GP workflow integration, system interoperability, and aligned incentives. Involving stakeholders early is essential to ensure that new digital tools meet the needs of both individuals with T2D and GPs.

## Introduction

General practitioners (GPs) worldwide face rising workloads, particularly in chronic disease management [1] and a declining workforce [2]. Among these conditions, type 2 diabetes (T2D) represents a major and growing challenge due to its increasing global prevalence and associated health care costs [3]. As primary care providers, GPs play a central role in managing T2D, yet high workloads can limit their ability to deliver optimal care [4].

These challenges are particularly pronounced in high-spending health care systems such as Switzerland, which ranks among the highest globally in per capita health care expenditure [5]. Despite this investment, approximately 6.1% of the Swiss population is affected by diabetes [6], contributing to a rising burden on primary care services. As diabetes prevalence continues to increase, GPs face mounting pressure to provide high-quality, long-term T2D management [7]. Together, these trends highlight the need for innovative approaches to better support GPs in managing T2D effectively.

At the same time, there is increasing evidence that structured monitoring and guideline-based diabetes management can contribute to improved patient outcomes. International studies suggest that patients who regularly receive recommended monitoring tests, such as glycated hemoglobin (HbA_1c_) measurements, lipid profiles, and kidney function assessments, have a lower risk of diabetes-related complications and hospitalizations [8]. Similarly, real-world evidence from Switzerland indicates that structured diabetes disease management programs can improve adherence to recommended monitoring processes while also slowing the growth of health care costs compared to usual care [9]. These findings highlight the potential value of systematic quality monitoring approaches in chronic disease management.

To support GPs, the Swiss Society of Endocrinology and Diabetology (SSED) developed the “SSED score,” a self-reported instrument to help GPs capture key clinical indicators, including HbA_1c_, blood pressure, and body weight during patient visits [10,11]. However, the SSED score is paper-based, making it susceptible to human error and limiting opportunities for longitudinal analyses and visualization of disease progression [12,13]. Moreover, the score lacks opportunities for data aggregation, automated scheduling of follow-up visits for individuals with T2D, and tailored lifestyle support. All these functionalities are increasingly relevant in modern long-term care [14].

Improving T2D management, therefore, requires a fundamental rethinking of how a quality assessment score is designed and implemented in practice. This includes not only the potential digitization but also integration into workflows, data infrastructure, and engagement processes. From a digital health adoption perspective, prior research suggests that the uptake of new technologies in clinical practice is influenced by perceived usefulness and perceived ease of use, as conceptualized in the technology acceptance model [15]. In primary care settings, perceptions of a quality monitoring score are likely shaped not only by the technical design of a tool but also by its compatibility with existing workflows [16] and its ability to reduce GP workload [17]. A key challenge is therefore how to improve quality assessment in a way that supports GPs in making more informed and efficient decisions while also addressing system-level (eg, workflow integration) constraints. To this end, we conduct a qualitative interview study to inductively explore stakeholder perspectives on digital quality monitoring for T2D management in Swiss primary care, with a focus on identifying challenges, limitations of existing quality assessment scores, and strategies to support their adoption in routine practice. We specifically examine three key research objectives (ROs):

1. RO1: identify current challenges in T2D management in primary care in Switzerland.
2. RO2: examine limitations of existing T2D quality assessment scores (eg, SSED score), including gaps in relevance, usability, and alignment with real-world care delivery.
3. RO3: develop strategies to promote the adoption of T2D quality assessment scores (eg, SSED score), addressing technical, organizational, and incentive-related factors to ensure a better fit with the complexities of chronic disease management in primary care.

## Methods

### Development of the Interview Guides

We designed semistructured interview guides tailored to each stakeholder group (refer to Multimedia Appendix 1). Each guide was structured around key thematic areas to ensure consistency across interviewees. At the same time, the format remained flexible, allowing interviewers to pursue unstructured, spontaneous questions where appropriate to explore emerging insights and context-specific experiences in greater depth.

### Selection and Recruitment of Interviewees

The interviews were conducted between November 27, 2024, and May 3, 2025. Health care professionals, health care software developers, and health insurers were recruited using purposive sampling. Participants were identified based on predefined criteria, including (1) professional involvement in T2D care, health software development, or health insurance decision-making; (2) experience with quality measurement or care coordination; and (3) relevance to the Swiss health care system. Potential participants were identified through the research team’s professional networks and publicly available information (eg, organizational websites and LinkedIn [Linkedin Corporation]).

Additionally, we used snowball sampling by asking the initial interviewees to suggest further participants who met our selection criteria, resulting in a chain of referrals.

Individuals with T2D were recruited via TestingTime (Norstad Group) [18], an online platform that compensates individuals for study participation. The inclusion criteria were that participants had been diagnosed with T2D, resided in Switzerland, and spoke either German or English.

Recruitment ended upon reaching theoretical saturation, defined as the point when 3 consecutive interviews revealed no new concepts [19,20].

A total of 38 qualitative interviews were conducted with 39 participants, representing 4 stakeholder groups relevant to digital T2D care: health care professionals (n=12), individuals with T2D (n=12), software developers (n=10), and health insurer representatives (n=5) (refer to Multimedia Appendix 2).

Most interviews were conducted on an individual basis. One interview with a health care software provider was conducted as a joint interview with 2 participants, resulting in a higher number of participants than interviews. Throughout the paper, participant counts (rather than interview counts) are reported to ensure consistency and transparency.

### Data Collection and Data Analysis

All interviews were conducted via video conference by 1 of 4 members of the research team (LJA, MP, OFG, or SM), all based in Switzerland. The interviewers were familiar with the study context. OFG had training and experience in conducting qualitative interviews and provided guidance and preparatory instruction to the other researchers before data collection, ensuring a consistent and structured interview approach. LJA conducted one interview with a software provider with whom she had prior professional contact, and SM conducted interviews with 3 GPs with whom he had prior contact. All remaining participants were not known to the interviewer who conducted the respective interview before the study. Interviews were conducted primarily in (Swiss) German (n=36), with 2 interviews conducted in English.

All interviews were digitally recorded via Microsoft Teams. Transcripts were generated using Microsoft Teams’ automated transcription function and were subsequently carefully reviewed by the research team. Any transcription errors or missing passages were manually corrected or added by relistening to the audio recordings before analysis. Transcripts were first analyzed and coded in German. Following the analytical process, codes and themes were translated into English for reporting purposes, with translation focusing on conceptual rather than literal equivalence. Given the systematic manual review and correction of transcripts before coding, automated transcription errors were considered unlikely to have materially affected the coding and interpretation of the data.

Next, transcripts were anonymized and assigned a unique participant ID. Data analyses were carried out in parallel using ATLAS.ti software (version 25.0.1; Lumivero, LLC). To ensure methodological rigor and transparency, we followed the COREQ (Consolidated Criteria for Reporting Qualitative Research) guidelines throughout the study (Checklist 1) [21].

Data were analyzed inductively to identify themes grounded in participants’ experiences and perspectives related to T2D management and digital tool adoption.

The research team’s positionality was shaped by backgrounds in computer science and business innovation, with a shared focus on digital health and health care innovation. Reflexivity was addressed at the team level through regular analytical exchange. Emerging findings, preliminary codes, and interpretations were systematically discussed and compared in weekly research meetings, allowing the team to reflect on differing perspectives, challenge initial assumptions, and iteratively align interpretations throughout the analysis process.

To enhance the rigor of the qualitative analysis, a subset of the interview transcripts was double-coded. Specifically, 6 of the 38 transcripts were independently coded by 2 researchers. Double-coding was conducted in 3 coding pairs: LJA-MP, SM-OFG, and LJA-OFG.

In an initial analytical step, separate preliminary codebooks were developed inductively for each stakeholder perspective to capture perspective-specific concepts and language reflected in the data. These first-order codes closely followed participants’ own terms and meanings. Discrepancies identified during double-coding were discussed within the respective coding pairs in iterative consensus discussions, which focused on clarifying code definitions and interpretations and informed refinements of the perspective-specific codebooks.

In a subsequent step, the perspective-specific codebooks were systematically compared and overlaid to identify overlapping concepts and recurring patterns across stakeholder perspectives. For example, issues related to fragmentation of care emerged consistently across multiple perspectives, albeit described using different terms. Based on these overlaps, first-order codes were progressively merged and abstracted into higher-level conceptual categories and themes.

LJA and OFG iteratively refined, consolidated, and validated the final set of codes and themes through discussion until the thematic structure coherently captured both cross-cutting and perspective-specific insights across the dataset.

In addition to the interviews, one follow-up clarification was obtained via email from an interview participant during the paper revision process. This exchange focused on a specific question related to anticipated changes in the Swiss billing system and was used to support contextual interpretation of the findings.

### Ethical Considerations

This study was reviewed by the institution’s ethics committee (HSG Ethics Committee), which determined that the project was exempt from formal ethics review (decision date: October 27, 2024). Written informed consent was obtained from all participants before participation in the interviews. All data were treated confidentially and processed in accordance with applicable data protection regulations. Interview data were anonymized before analysis, and no directly identifiable personal information was included in the study results. Data were stored securely on institutional systems accessible only to the research team. Individuals with T2D who were recruited via TestingTime received financial compensation of CHF 20 (US $25.53) for their participation, in accordance with the platform’s standard reimbursement procedures. Health care professionals, software developers, and representatives of health insurers did not receive financial compensation.

## Results

### Overview

We conducted a total of 38 individual interviews, with the coding framework described in Tables 1-4. Interviews were structured in 3 main parts. In the first part of the interview, participants were asked about their current challenges in T2D management (RO1). In the second part, they were asked about the limitations of existing T2D quality assessment scores (eg, SSED score; RO2). In the third part, participants discussed recommendations to address these limitations through actionable strategies. These strategies aimed to promote the adoption of T2D quality assessment scores (eg, SSED score), considering technical, organizational, and incentive-related factors and the complexities of chronic disease management in primary care (RO3).

**Table 1.** Themes and codes for the status quo and key challenges in primary care.

Themes	Codes
Systemic and structural burdens	Communication with other health care providers Absence of holistic care Time pressure Shortage of skilled workers
Lifestyle and personal challenges	Difficult maintaining a healthy diet Low motivation for exercise Being in denial about the disease
Primary care challenges	Late or unexpected diagnosis Insufficient and impersonal information Absence of holistic care Limited clinic availability
Structural and technological barriers	Financial constraints Access barriers to sports facilities Limited data sharing of vital parameters

**Table 2.** Themes and codes for guideline adoption barriers.

Themes	Codes
Monetary and nonmonetary rewards	Limited ability to decide how to use financial rewards Only consultations are compensated, not administrative tasks The reach of incentive programs is low Exclusion of noncompliant patients Nonmonetary rewards are not perceived as valuable
Fragmentation and interoperability	Fragmentation of guidelines Fragmentation in digital infrastructure and a lack of system interoperability Manually re-enter health data Inadequate quality of data entry
Tension between guidelines and real-world practice	Perceived lack of value in guidelines, as they do not necessarily reflect the quality of care Score components depend on patient adherence GPs^a^ resist transparency in benchmarking performance Patients do not have diabetes alone, but from multiple chronic conditions
Regulatory barriers	Strict regulations regarding patient data (Switzerland) Classification as a medical device

a GP: general practitioner.

**Table 3.** Codes for essential features for the Swiss Society of Endocrinology and Diabetology (SSED) score.

Themes	Codes
Reducing structural burdens	Integration or compatibility with clinical information systems Integration of patient data
Targeted digital interventions	Labeling and flagging system Reminder and alert mechanisms Multidisease scores Inclusion of patients with prediabetes
Aligning with patient needs	Collaborative goal setting Customizable patient score

**Table 4.** Codes for adoption strategies for the Swiss Society of Endocrinology and Diabetology (SSED) score.

Themes	Codes
Task shifting	Involving MPAs^a^, MPKs^b^, and APNs^c^
Aligning financial incentives with quality care	Financial incentives Fund continuing education Replacement of the outdated TARMED^d^ system
Boosting communication and data sharing	Improved communication Sharing and comparing data Feedback loops Automated solutions to support decision-making
Leveraging digital tools to support clinical decision-making	Automated solutions to support clinical decision-making
Strengthening patient support	Updated and personalized information Collaboration between GPs^e^ and pharmacies

a MPA: medical practice assistant.

b MPK: medical practice coordinator.

c APN: advanced practice nurse.

d TARMED: tarif médical.

e GP: general practitioner.

### Key Challenges With T2D Management in Primary Care

This section outlines the key challenges of T2D management (RO1).

#### Systemic and Structural Burdens

In typical diabetes consultations, GPs often manage individuals with T2D with multiple comorbidities within limited timeframes, relying heavily on support staff. Six of 10 (60%) GPs mentioned that they occasionally rely on the support of endocrinologists to ensure high-quality care. Furthermore, 5 of 12 individuals with T2D (~42%) were unhappy with the absence of holistic care. They felt that GPs addressed only one issue at a time, with little coordination between providers: “[…] everything is an isolated system.” (P8)

Five of 12 (~42%) health care professionals reported experiencing constant time pressure in their daily practice. With the next person typically waiting, in-depth discussions or more intensive consultations become difficult to manage. As a result, GPs often struggle to provide the level of attention and care they would ideally like to offer. GP2 stated: “Initially, it is certainly the case that people with diabetes require a great deal of effort if you want to inform them properly…. Instead, we refer them to hospitals, which have diabetes counseling services and dietetic counseling units, because we cannot afford this time investment. Endocrinologists have more time, and they are also able to bill this very well. We, on the other hand, are not.”

Additionally, 3 of 12 (25%) GPs noted a growing shortage of skilled workers, whereby medical practice assistants (MPAs) and medical practice coordinators (MPKs) are increasingly difficult to recruit, as mentioned by GP7: “It has become quite difficult, and I believe even in larger centers it has become challenging in some cases. It is not yet as severe as it is for physicians, but MPAs are in high demand.” Furthermore, it was mentioned that even when MPAs are available, they often do not remain in the same practice long enough to complete further training. As a result of this shortage, other health care professionals are forced to take on additional responsibilities, particularly administrative tasks.

#### Primary Care Challenges

Five of 12 (~42%) individuals with T2D reported having a generally trusting relationship with their GP. However, 6 of 12 (50%) individuals with T2D stated that the diagnosis was late or unexpected. Participants were often diagnosed during unrelated check-ups or procedures, or after requesting further testing themselves. This may reflect not only delayed diagnosis but also insufficient preventive guidance or poor advice by the GP—for example: “I wanted to do a hair transplant […] and then suddenly the doctor mentioned that I have type 2 diabetes” (P6), and “My GP never told me to change my lifestyle habits although I was always at the upper limit […] I could have taken better care of myself earlier” (P2). Also, 7 of 12 (58%) interviewees said that after their diagnosis they received insufficient and nonpersonalized information, which limited individuals with T2D's ability to manage their condition. The individuals with T2D described receiving outdated or overly generic advice from GPs that might have been used in diabetes education 20 years prior. Dietary guidance was also seen as insufficiently personalized: “I also have grain intolerance and a dairy intolerance […] I really noticed that the nutritionist was desperate. She couldn’t apply any standard approach” (P5). New technologies, such as continuous glucose monitors, were rarely mentioned or explained by GPs. Limited clinic availability presented additional barriers. Three of 12 (25%) individuals with T2D reported long waiting times and inflexible hours, making routine consultations hard to access. P8 stated: “I have to go on an empty stomach […] I always have to take vacation days for the GP visit […] that’s why I’m going only 1‐2x per year” (P4).

#### Structural and Technological Barriers

Four of 12 (~33%) individuals with T2D reported that frequent blood sugar measurements helped them become more disciplined about their dietary choices. They mentioned, however, that financial constraints hindered them from ideal self-management, especially due to the cost of test strips: “One test strip costs almost 1 franc. If you want to test regularly, it gets really expensive” (P2). Three of 12 (25%) individuals with T2D said that they faced access barriers to athletic facilities. One participant lost access to Aqua Gym when it closed during the COVID-19 pandemic, while others found traveling to fitness centers too difficult and time-consuming: “The gym is too far […] so I rather stay at home and go for walks sometimes” (P12). Seven of 12 (~58%) interviewees used some digital device (diabetes app, smartwatch, step tracker, continuous glucose monitor, blood pressure device, and other) to measure their vitals frequently. Four of 12 (~33%) individuals with T2D reported that sharing their vitals with GPs was not possible due to data-sharing issues. They manually consolidate and print data for consultations: “So I print the blood pressure data from the app and bring it to the doctor. It’s just extra effort that is tedious” (P1). Only one individual with T2D said that their endocrinologist has direct access to their continuous glucose monitoring device data.

### Barriers to Adopting T2D Quality Assessment Tools Such as the SSED Score

This section explores the key barriers that hinder the adoption of T2D quality assessment tools, such as the SSED score (RO2).

#### Monetary and Nonmonetary Rewards

The current system compensates for consultations, but not for administrative tasks, such as those related to the SSED score. This contributes to a structural issue, whereby physicians are compensated based on the quantity of consultations rather than the quality of care provided: “The processes can be made more efficient, and I also believe they can be evaluated and used as a quality indicator—or even incentivized” (PT1). Four of 5 (80%) insurers acknowledged that they have limited ability to incentivize practices beyond standard reimbursable services, such as administrative documentation or coordination.

In contrast, 4 of 12 health care professionals (~33%) reported using the SSED score in their practice as part of managed care contracts with health insurers. In these arrangements, health insurers give financial incentives to the managed care network, which then decides how to allocate those funds. As a result, individual providers have limited voice in deciding how these financial resources are used. One GP mentioned that their network uses this compensation to organize workshops and invest in further education for both the GP and the team to improve the quality of T2D management: “Through our managed care contracts we have negotiated with the health insurers that if we offer cost-effective, good quality, our network can use some of the money for training and projects” (GP1).

However, insurers noted that the reach of these incentive programs remains low due to variability and fragmentation across insurance plans: “Reaching a large population is much more challenging because [A] these products vary across insurers, and [B] the number of people covered by these specific products is usually quite low” (HI3). Additionally, 1 of 5 (20%) health insurers pointed out that some medical practices may exclude noncompliant individuals with T2D to improve their overall SSED score and qualify for financial incentives. This selective reporting can distort the actual quality of care: “Whenever we gave someone a score like that, most of them always achieved it. They just didn’t include the people who didn’t fit in” (HI3).

Finally, 5 of 12 (~42%) health care professionals noted that nonmonetary rewards, such as performance ratings, awards, or labels, are not as interesting and, in some cases, unwanted. One GP mentioned that practices are already at full capacity, making such labels irrelevant for attracting more individuals with T2D: “I don’t want all diabetics to come to me because I’m somehow labeled as such. That really doesn’t add any value from my point of view...I’m not an endocrinology practice.” (GP7), while GP8 noted: “At the moment, we have almost 20 patient rejections per day in our practice. People are simply glad if they can find care anywhere, and a label like this brings no added value for me at all.”

#### Fragmentation and Interoperability

Health care professionals consistently emphasized their reliance on T2D guidelines but also expressed frustration with their fragmentation. These guidelines originate from various organizations, often resulting in inconsistent benchmarks and uncertainty about best practices.

All 12 (100%) health care professionals also reported using a clinical information system (CIS) to manage individuals with T2D, not only for diabetes but across their practice. However, they highlighted fragmentation in digital infrastructure and a lack of system interoperability as significant challenges.

Five different CISs were mentioned, some of which include diabetes-related functions, such as targeted monitoring, while others serve only basic documentation purposes. While 7 of 10 (70%) health care software developers offer diabetes dashboards or SSED-based tools, these are rarely integrated into practice software systems. Instead, they are accessed via separate web portals or stand-alone apps that require GPs to log in outside their primary clinical workflow. This was highlighted in the following way: “The practice software providers are not necessarily known for offering interfaces, or if they do, they are very expensive. […] The market is currently very fragmented, with around 60 different primary CIS” (SP1). Due to missing or inadequate interfaces, many providers reported needing to manually re-enter health data that was already stored in the CIS. Seven of 10 (70%) GPs stated that this not only wastes their time but also discourages them from using digital tools: “I also think that the entire bureaucracy in medicine is too much and if you then have to fill out something more, it is simply an additional burden” (SP9).

A key concern of health insurers is the inadequate quality of data entry in CISs: “The quality of data capture is often inadequate, and data is also lost when transferred to the SSED score” (HI4). Three of 5 (60%) insurers emphasized that unstructured data within CISs poses a significant obstacle to developing reliable interfaces. Compounding this issue is the predominance of unstructured and text-based data in many CISs, as noted by 4 of 10 (40%) health care software developers: “Often certain information is extremely difficult to read and is then either written as free text or not recorded at all” (SP5).

#### Tension Between T2D Quality Assessment Tools and Real-World Practice

Five of 12 (~42%) health care professionals argued that quality assessment scores, such as the SSED score, do not bring any value, as they do not necessarily reflect the quality of care provided: “I don’t think that the SSED score per se helps to improve processes. The SSED score simply tells you how well I look after my team. It doesn’t help with the processes” (GP4). The potential risk of false-positive outcomes when working with the SSED score was also highlighted: “So I can cause a falsely good SSED score if I simply tick a box but only quickly look at the foot and have not examined it properly, for example. The examinations, in particular, are parameters that allow for some margin and do not necessarily reflect the true quality” (GP9). Such false-positive results may arise when certain parameters, such as the foot examination, are marked as completed despite being carried out superficially or interpreted with significant flexibility. Thus, 2 of 12 (~17%) health care professionals emphasized that it is possible to improve the SSED score by selectively entering data. This can then lead to an inaccurate portrayal of treatment quality.

Furthermore, health care professionals emphasized that achieving a good SSED score in everyday practice is often difficult. Four of them (~33%) pointed out that many score components depend on patients’ adherence or factors beyond the physician’s control, leading to potential false negatives, such as elevated HbA_1c_ levels despite appropriate treatment. GP1 mentioned: “There are people who want to take a great deal of responsibility themselves and are very well informed; they come prepared with questions. Others, however, are already overwhelmed from the very beginning. A classic example, just as an anecdote: people know that diabetes is a “sugar disease,” but I would say that about one third of patients never fully understand that it is not only about sugary foods—100 grams of dry spaghetti are essentially equivalent to 99 grams of pure sugar.” Six of 12 (50%) health care professionals highlighted that inconsistent self-management is a key barrier, as effective T2D care requires sustained lifestyle changes, which some individuals with T2D struggle to maintain. Health insurers echoed this concern. They stressed that many SSED score components rely directly on patients’ behavior, which is influenced by broader social and economic conditions: “We also know that, especially with these chronic illnesses, socioeconomic factors come into play that the family doctor cannot change per se. A lot of it is about financial possibilities, but also level of education or the cultural environment in which the patient lives. That definitely has an influence” (HI1). Insurers also noted that poor self-management contributes not only to worse outcomes but also to increased health care costs, highlighting the need for a more interdisciplinary and socially informed approach to T2D care.

All 5 (100%) health insurers stressed that a lack of openness and consensus among health care professionals hampers effective management of T2D. Many GPs resist transparency when it comes to benchmarking performance: “Transparency can be uncomfortable for doctors because they are not used to seeing their own results in a benchmark” (HI5). Four of 5 (80%) health insurers also mentioned that GPs often assume they are already managing their individuals with T2D well, making it difficult for them to accept that their care might fall short in certain areas.

Eight of 12 (~67%) health care professionals noted that individuals with T2D rarely have diabetes alone and often have multiple chronic conditions: “Many individuals with T2D have additional diseases. This can make the treatment difficult” (GP6). These individuals with T2D often require highly individualized and interdisciplinary care approaches. Multimorbid cases challenge the standardization of treatment plans and demand greater flexibility in clinical decision-making. In this context, 4 of 12 (~33%) health care professionals argued that digital solutions can be difficult to integrate, as they often lack the necessary usability to handle such complex cases. In this regard, all 5 (100%) health insurers highlighted that long-term conditions need an interdisciplinary approach involving GPs, specialists, MPAs, and others.

Also, 6 of 10 (60%) health care software developers emphasized that diabetes management requires input from multiple disciplines and diverse reasoning approaches, making the integration of a standardized digital solution complex: “Multifactorial means that many aspects have to be taken into account when it comes to the therapy or treatment of diabetes” (SP3). This complexity underscores the challenge of developing a comprehensive digital solution that integrates relevant medical, lifestyle, and treatment data.

#### Regulatory Barriers

Six of 10 (60%) health care software developers identified strict Swiss regulations with health data, particularly those related to billing codes and health data documentation, as key obstacles: “The documentation requirements are strict, and there are many requirements regarding retention obligations and data protection” (SP3). Finally, 4 of 10 (40%) health care software developers highlighted the significant burden imposed when an application is classified as a medical device. Such a classification entails extensive certification processes, high costs, and stringent regulatory requirements that must be continuously met, reevaluated, and updated: “We did the math and came up with a multi-million-dollar figure, which is not feasible for us” (SP9).

### Suggestion to Adopt T2D Quality Assessment Tools

The following section summarizes suggestions for improving the SSED score to better support GPs in T2D management. It highlights essential features the tool should include and outlines practical strategies to encourage its adoption in routine GP practice (RO3).

#### Essential Features

We asked the participants to identify the key essential features for an application necessary for successful management of T2D, specifically related to the SSED score. The results focus on reducing structural burdens, improving T2D management, and aligning with GP needs.

##### Reducing Structural Burdens

A key requirement highlighted by 5 of 12 (~42%) health care professionals, 5 (50%) health care software developers, and 5 (100%) health insurers is that the SSED score application must be integrable or compatible with existing CISs. As a health care software developer put it, “A connection to the CIS would be ideal” (SP9), and a GP emphasized, “The most important thing is that such features are fully available within the practice information system” (GP4). Further, GP2 reported: “There would certainly be a benefit when the scores were somehow compatible with standard practice software and could automatically extract the necessary data.” Furthermore, 6 of 12 (50%) health care professionals stressed the importance of directly incorporating patient-reported data into the application, rather than relying on individuals with T2D to hand over PDF documents during consultations: “It’s crucial to have a fully integrated solution or embed these data-gathering steps into the consultation itself so they can be reviewed in real time” (GP4). Four of 12 (33%) individuals with T2D shared this view. They would appreciate a unified digital platform to track all health data, such as glucose, blood pressure, medication, and so on and to sync it with their GP records. Visualizing long-term trends was seen as a valuable tool for managing their condition. One individual with T2D shared: “I’m setting up the electronic health record (EHR). Ideally, I could submit all my data to a single platform and get an overview” (P6). However, 1 of 10 (10%) health care software developers cautioned that true interoperability remains a major barrier, emphasizing that without a functioning EHR infrastructure in Switzerland, full integration will not be possible.

##### Targeted Digital Interventions

All 10 (100%) health care software developers emphasized the need for a robust labeling and flagging system that categorizes individuals with T2D based on key metrics, highlighting critical challenges in managing T2D. This approach allows care teams to quickly identify individuals with T2D who are struggling and prioritize interventions: “It’s valuable to compare individuals with T2D. Who’s doing well and, more importantly, who isn’t? Why? Are we missing something, or are their values genuinely poor?” (SP9) Two of 5 (40%) health insurers echoed this requirement. In addition to labeling and flagging, 8 of 10 (80%) health care software developers highlighted the importance of a reminder or alert mechanism that automatically notifies health care professionals or individuals with T2D when critical thresholds are reached. Such reminders ensure that high-risk individuals with T2D receive timely follow-ups, whether consultations, laboratory tests, or medication reviews, so that no essential checkups are overlooked.

Three of 5 (60%) health insurer representatives highlighted the need to develop multidisease scores that can be accessed through digital solutions. One of 5 (20%) health insurer representatives suggested creating a solution that extends beyond the SSED score to include other chronic disease scores, such as the CARE score [22] (a cardiovascular prevention score). Five of 10 (50%) health care software developer interviewees emphasized that applications should not only support individual scores but also focus on population health: “For us, diabetes is just the beginning. In the long term, the system can and should evolve—such as for multimorbidity indicators or other relevant measurements” (SP3). SP2 mentioned: “Once we reach this level (chronic disease management), we would essentially have conditions comparable to those in modern, well-digitalized health care systems—often cited examples include Denmark, Finland, and Sweden. In such systems, these prerequisites already exist. Under certain circumstances, this could represent a real breakthrough.”

Finally, 3 (30%) health care software developers suggested including individuals with prediabetes in digital diabetes management applications: “This raises the question of whether a minimal version of the SSED guidelines could be developed specifically for prediabetics” (SP2). Including individuals with prediabetes would help identify and manage risk factors early, potentially preventing or delaying the onset of T2D.

##### Aligning With Needs

Two of 5 (40%) health insurers emphasized that setting goals collaboratively with individuals with T2D could improve adherence. Similarly, 2 of 10 (20%) health care software developers suggested that GPs should be able to define specific, personalized goals with individuals with T2D to better identify issues and set realistic, achievable targets. This approach also provides individuals with T2D with individualized benchmarks to work toward daily, promoting awareness and increasing adherence. In this context, 2 of 12 (16%) individuals with T2D highlighted the need for a digital solution that actively supports them through tailored feedback and education. A digital SSED application could therefore link individual score results to personalized, evidence-based educational content (eg, short explanations or practical recommendations) that explain the relevance of indicators such as HbA_1c_, blood pressure, or weight in an understandable way. This need was explained in the following way: “Like, if you look at the UK, the NHS has a separate department for diabetes, where they really put out a lot of information very frequently and they’re updating their knowledge. (..) For example, you can also look up the coaching platform that I use: Mastering-Diabetes.org, nutritionfacts.org. These are very helpful resources that don’t exist in Switzerland” (P8).

Four of 12 (33%) health care professionals argued that the SSED score requires a more flexible interpretation, as its current rigidity can hinder practical implementation: “Because it is impossible to achieve this 80% rate, we adopted the SSED score criteria, but we completely changed the interpretation to be able to achieve reasonable care” (GP6). Such adaptations reflect the need for scoring systems to accommodate everyday practice realities rather than impose idealized standards. Furthermore, 1 of 10 (10%) health care software developers emphasized that an SSED score–based application should offer customizable scoring features tailored to specific practice workflows and populations: “The way doctors handle management varies greatly from practice to practice. I’m talking more about a workflow here—some would like it this way, others would like it that way” (SP10). This perspective was echoed by 1 of 12 (8%) health care professionals, reinforcing the need for flexibility and configurability in digital tools to support meaningful, context-sensitive care delivery.

### Guideline Adoption Strategies

In addition to exploring key problems in T2D management and essential features for an application, we asked the interviewees which strategies they consider essential for improving the adoption of the SSED score and enhancing the overall management of T2D.

#### Task Shifting

Eleven of 12 (~92%) health care professionals emphasized the important role of MPAs, MPKs, and advanced practice nurses in diabetes management, to whom physicians can delegate specific tasks and consultations: “They help us a lot. It’s increasingly being divided so that we, doctors, handle part of the four examinations, and the MPA does the other part—two and two, for example” (GP10). Additionally, 3 (30%, 6/10) health insurer representatives and 6 of 10 (60%) health care software developers agreed that involving these health care professionals could improve diabetes care by allowing GPs to delegate responsibilities, including administrative tasks.

#### Aligning Financial Incentives With Quality Care

Ten of 12 (83%) health care professionals emphasized that financial incentives could serve as a strong motivator. Five of 12 (42%) health care professionals highlighted the following: “I think the effort should be rewarded—the time you put in. And of course, if you put in more effort, you should get something back for the time you invest” (GP3). One of 12 (8%) health care professionals also suggested that such compensation could be used to fund continuing education, workshops, and training initiatives that directly contribute to improved diabetes care. This perspective was echoed by 2 of 5 (40%) health insurers, who placed hope in the upcoming TARDOC (Tarif für ambulante ärztliche Leistungen und Dokumentation) pricing structure, set to replace the outdated TARMED (tarif médical) system in 2026. TARDOC is designed to modernize the compensation model, aligning it more closely with today’s medical practices and technologies. It aims to ensure fairer remuneration for services rendered while reducing inefficient incentives and promoting value-based care within the health care system: “I think there’s hope that with TARDOC, we’ll have a model that’s a bit more flexible and innovative. That we can also ensure that certain services can be financed, which are important but are currently difficult to include in existing models” (HI3).

#### Boosting Communication and Data Sharing

A recurring theme among interviewees was the need for improved communication and data sharing within diabetes care networks. Nine of 12 (75%) health care professionals believed that better communication and collaboration between GPs and specialists could significantly improve the management of complex, multimorbid individuals with T2D. GP8 mentioned: “What would be the most effective and best tool would be a reporting and information exchange tool with hospitals—specifically for sharing clinical reports. Inselspital has implemented this, and Epic has now set it up for GPs: a login access that allows GPs to log into Epic and view reports, progress notes, and the full medical records of their own patients. That is, of course, excellent. If other provider groups were to implement something similar, it would significantly reduce uncertainties about what has happened or what is currently going on, because I could simply retrieve the information myself. This is, for example, a tool that I currently only know from Epic, and one that could be optimized and scaled substantially.”

One of 5 (20%) insurers shared a similar view, emphasizing the value of smooth, timely transitions to specialized care: “There’s long-term cost-saving potential, especially if GPs are supported in identifying which individuals with T2D should be referred and when” (HI1). Five of 10 (50%) health care software developers also stressed the importance of network-wide data sharing and comparison: “We have a benchmark in the network, so the doctor can see how they compare to other practices. It acts as a trust indicator” (SP4). These comparisons typically involve quality indicators and clinical outcomes (eg, HbA_1c_ levels and blood pressure control), allowing health care professionals to recognize areas for improvement and drive better care outcomes. While cost data were less frequently mentioned, the emphasis was on performance transparency across practices.

Four of 5 (80%) health insurers echoed this by recommending tools such as feedback loops and “improvement clubs” to promote transparency and continuous learning: “Once we create transparency and see how they compare, it opens up opportunities for ongoing improvement. It’s about using data, learning from it, making changes, and moving through a continuous learning cycle—Plan, Do, Check, Act” (HI1). This approach not only improves individual practices but also fosters a culture of shared responsibility and peer-driven quality enhancement across the network.

#### Leveraging Digital Tools to Support Clinical Decision-Making

Besides this, seven health care software developers (70%, 7/10) emphasized that automated solutions could support clinical decision-making through generating valuable insights to assist GPs. These technologies also hold promise for reducing administrative workload over time: “I think AI will have a revolutionary impact in both administration and medicine. It will take a lot of work off doctors’ hands and can create treatments tailored to the patient” (SP10). Additionally, five health care professionals (~42%, 5/12) acknowledged the potential of decision-supporting solutions to enhance and streamline medical decision processes.

#### Strengthening Support for Individuals With T2D

Several interviewees mentioned that the SSED score should be designed not only for GPs but also for other stakeholders. Three of 12 (25%) individuals with T2D called for closer collaboration between GPs and pharmacies. This could be implemented, for instance, through testing HbA_1c_ at pharmacies during off-peak hours and discussing results with GPs over the phone. This collaboration would improve access and flexibility in managing T2D: “It would be helpful if I could test my blood at the pharmacy and they send the results to my GP so we could discuss them over the phone” (P4). In this case, not only the GP but also pharmacists would have access to the SSED score–based application. This would alleviate the burden on GPs to fill out the SSED score themselves. On top of that, 9 of 12 (75%) individuals with T2D highlighted that if a SSED score–based application had a user interface, it should show updated and personalized information. Some proposed dedicated platforms or paper-based periodical publications with up-to-date content tailored to individual needs: “In the UK, the NHS puts out frequent updates and holds conferences” (P9). Five of 12 (~42%) health care professionals echoed this sentiment and noted that individuals with T2D are more likely to follow treatment plans when they understand their condition better: “If I know that individuals with T2D see the added value and the improvement, they are, of course, a lot more motivated” (GP8). Tailored information can thus play a key role in strengthening adherence and long-term management of T2D.

## Discussion

### Principal Findings

#### Overview

This study explored stakeholder perspectives (health care professionals, health care software developers, individuals with T2D, and health insurers) on challenges in T2D management in Swiss primary care, the limitations of the existing SSED quality score, and strategies to support its adoption in routine practice. Findings indicate persistent structural barriers, including time pressure for health care professionals, fragmented care pathways, and limited personalized support for individuals with T2D. Stakeholders also identified obstacles to digital quality monitoring, particularly poor integration with GP systems, misaligned incentives, and limited suitability for multimorbidity. At the same time, facilitators of adoption included workflow integration, task shifting, patient-centered functionality, and improved data sharing. Overall, the results suggest that successful implementation of a digital SSED score requires addressing organizational, technological, and behavioral factors across the care system. Although the SSED score is specific to the Swiss T2D context, the underlying principles may also be relevant for other chronic conditions and health care systems.

One of the most critical and unresolved challenges in Switzerland is the fragmented and unstructured nature of health care data. This challenge poses a fundamental barrier to the implementation of interoperable systems. Interviewees across stakeholder groups, particularly health care software developers and health insurers, consistently pointed to the lack of standardized interfaces and structured data as a core impediment. CISs are highly fragmented, often relying on free-text entries, manual exports, and redundant documentation processes. Even the most well-designed digital SSED score cannot unlock its potential if data from various sources, such as individuals with T2D, pharmacies, devices, or laboratories, cannot be aggregated and meaningfully connected.

This lack of integration is not a minor technical issue but one of the defining barriers. It prevents automation, limits decision-support capabilities, and undermines the utility of performance feedback. Therefore, the challenge of bringing together disparate, siloed, and often poorly structured data is a technical prerequisite and represents the most urgent priority for digital transformation in long-term care. Without addressing this foundational issue, the envisioned digital support systems risk remaining conceptual rather than operational.

#### Essential Features

Findings from the interviews show that for the SSED score to be adopted at scale, it must be embedded into the daily workflows of primary care in a way that minimizes administrative burden while maximizing clinical utility. A core requirement is interoperability. Data entered by individuals with T2D or MPAs must seamlessly synchronize with the GPs’ EHRs, such as the CIS. This ensures that information is not siloed and that GPs maintain a complete, up-to-date overview without duplicating effort.

These findings are supported by existing studies. For example, it has been shown that individuals with T2D treated in hospitals with advanced EHRs cost nearly 10% less than those in hospitals without advanced EHRs, due to better care coordination, fewer duplicative tests, and increased efficiency [23].

Furthermore, a recent literature review showed that EHRs with predictive analytics can anticipate disease progression, support earlier intervention, and improve patient outcomes, particularly in conditions such as diabetes and cardiovascular disease, while also reducing hospitalizations and health care use [24].

Furthermore, our findings show that the user interface of the SSED score must also be intuitive and time-efficient. Quick data entry fields, automated alerts for missing or abnormal values, and visual dashboards summarizing an individual’s status (eg, traffic-light systems) can support clinical decision-making while saving time. To reconcile standardized quality assessment with individualized care, the authors suggest that the digital SSED score should be designed as a modular system. This is particularly important for individuals with multimorbidity.

A modular structure could enhance usability. In such a design, individual sections of the score (eg, HbA_1c_, blood pressure, and weight) can be completed independently and at different times. This allows clinicians to gradually fill in the score during multiple visits while the system tracks overall completion status.

The authors suggest that such a modular architecture would enable the integration of additional disease-specific or cross-disease quality metrics, such as multidisease scores, without overburdening clinicians. From the authors’ perspective, this approach would allow a digital SSED tool to preserve a standardized core set of diabetes indicators while flexibly expanding to capture comorbid cardiometabolic conditions, thereby supporting holistic, patient-centered care in multimorbidity contexts.

Based on the study findings, the authors further recommend empowering individuals with T2D as active contributors by integrating home-monitoring devices (eg, blood pressure monitors and wearables) and allowing manual input of parameters such as weight, blood pressure, or step count. Incentive mechanisms and nudges, such as accomplishment badges and reminders, could further increase engagement.

Prior research suggests that patient-facing applications that support self-monitoring and provide feedback can improve engagement and health outcomes, particularly in chronic disease management [25]. Features such as reminders, goal tracking, and gamification elements have been found to enhance motivation and adherence to self-care behaviors [26,27].

#### Adoption Strategies

##### Overview

While financial incentives such as pay-for-performance schemes have been proposed to enhance chronic disease management [28], our interviews reveal mixed perceptions of these schemes. Although GPs indicated that receiving additional compensation for completing the SSED score would be appreciated, they also noted that merely filling out the score may not necessarily lead to improved clinical outcomes. Moreover, if payment were tied to clinical outcomes, health insurers raised concerns that GPs may selectively document only well-managed individuals with T2D in the SSED score to protect their performance metrics. While financial incentives could be powerful motivators for GPs, they must be carefully designed to avoid discouraging the inclusion of complex or noncompliant individuals with T2D.

Based on additional literature, Helsana, one of Switzerland’s largest health insurers, successfully piloted a pay-for-performance model that resulted in a 14% increase in guideline-adherent diabetes care; however, critics argue that it remains unclear whether individuals’ clinical outcomes improved [29].

Our findings further showed that certifications were also seen as an unappealing incentive for GPs to use the SSED score, as many are already managing full practices and are not seeking to attract additional individuals with T2D. Instead, time-saving approaches and task redistribution emerged as more promising strategies to foster the implementation of the SSED score. Three potential solutions were identified as feasible and contextually relevant.

##### Task Shifting to MPAs With Specific Diabetes Education

Our findings indicate that MPAs are already engaged in routine clinical tasks such as blood sampling and education of individuals with T2D. Expanding their scope of practice to include SSED score completion, especially if supported by additional training in diabetes care, could reduce GPs’ workload while enhancing the quality of interactions with individuals with T2D. MPAs are not only more cost-effective than physicians but also have the potential to engage more deeply with individuals with T2D, thereby supporting better adherence and health outcomes.

Evidence from a Swiss case report further illustrates that when individuals with T2D are given space to openly discuss their medication concerns through active listening, they can move from nonadherence to sustained adherence, highlighting the importance of communication skills alongside clinical delegation [30]. Furthermore, a recent meta-analysis shows that, compared with standard care, models in which nurses take a leading role achieve meaningful improvements in clinical indicators of diabetes and hypertension [31].

A Swiss study evaluating the long-term implementation of a care program, primarily delivered through specially trained practice nurses, showed significant improvements in HbA_1c_. However, only 40.9% of practices continued using this model 3 years after the initial intervention. Key barriers included financial constraints, such as the lack of reimbursement for practice nurses, and a reluctance among GPs to delegate clinical responsibilities [32].

One health insurer (HI1) noted that TARDOC, expected to be implemented in 2026, introduces only incremental changes for nonphysician chronic care activities. Similar to TARMED, it includes chronic care management positions (AK.05) that allow services delivered by MPAs or MPKs to be billed, while shifting from 15-minute blocks to minute-based accounting and expanding eligibility to additional chronic conditions. Consequently, the economic viability of delegating structured diabetes management to MPAs or MPKs depends largely on practice-level efficiency gains, such as freeing physician capacity for higher-value services, rather than on tariff reform alone.

##### Involving Pharmacies in Routine Monitoring

Although we did not interview pharmacists, interviewees in our study suggested that pharmacies could be a helpful resource in chronic disease management.

It is plausible that shifting routine diabetes monitoring—such as HbA_1c_ or cholesterol checks—from GPs to pharmacies could offer economic and logistical advantages. A pharmacy-based HbA_1c_ test costs around CHF 30 (US $38.28) [33]. Adding a teleconsultation with a GP (CHF 50 [US $63.79]) [34] could bring the total to CHF 80 (US $102.07)—less than half the cost of a standard GP visit (CHF 150 [US $191.38]) [35]. For well-controlled individuals with T2D, 2 annual pharmacy visits combined with 1 in-person GP check (eg, for nephropathy, retinopathy, and foot exams) could maintain care quality while substantially lowering costs. Findings from the Swiss Siscare program, a pharmacy-led adherence program, further support this potential: pharmacists effectively conducted medication adherence monitoring and counseling. However, collaboration with physicians was often limited to one-way information flows, underscoring the importance of interoperable IT systems and clear reimbursement models for sustainable implementation [36].

From the authors’ perspective, this development could be transformative for the SSED score: pharmacies could act as local hubs for routine monitoring and structured data entry, thus alleviating GP workload and enhancing continuity of care. The model also aligns with broader goals of cost efficiency and prevention, as better medication adherence and early interventions have been shown to reduce complications [37,38]. However, as previous studies show, successful implementation will depend on the integration of interoperable systems, well-defined roles, and close coordination among health care professionals [39,40].

##### Empowering Individuals With T2D Through Self-Tracking and Data Sharing

Based on the interviews, some individuals with T2D already monitor health parameters using wearables and expressed a willingness to share these data with their GPs.

Building on this insight, the authors propose that individuals with T2D could additionally complete laboratory-based metrics (eg, HbA_1c_ and cholesterol) in pharmacies, thereby creating a comprehensive, longitudinal health profile accessible to GPs. Such a model might support more personalized care and greater engagement and accountability. It also allows health insurers to experiment with value-sharing schemes, such as rewarding data-sharing individuals with T2D with bonus programs or lower premiums. In Switzerland, such models are common; health insurers have introduced apps that promote healthy lifestyles by offering monetary rewards for sharing behavioral data from fitness trackers or manual inputs [41]. From the authors’ perspective, if implemented effectively, this triadic model, combining individuals’ self-tracking, pharmacy-based services, and GP oversight in one digital solution, could offer a scalable, cost-saving solution that maintains high standards of care.

These findings suggest that meaningful SSED score adoption will require more than a single incentive mechanism. A multipronged approach, grounded in interprofessional collaboration, digital enablement, and empowerment, holds greater promise. Importantly, the upcoming regulatory changes in Switzerland provide fertile ground for the trial of such models. Yet, the success of these strategies will depend on several enabling factors: interoperability of digital systems, provider trust in redistributed responsibilities, and robust evidence demonstrating the cost-effectiveness and clinical utility of these alternative pathways.

### Practical Recommendations and Future Directions

Across all stakeholder groups, the findings consistently indicate that resolving data infrastructure fragmentation is the primary prerequisite for any scalable digital enhancement of T2D quality measurement. Without interoperable data flows and shared data standards, advanced digital features cannot function reliably or deliver value at scale. Taken together, these findings suggest several core design principles for future digital health tools: (1) interoperability should be treated as a foundational requirement rather than an optional feature; (2) systems must integrate seamlessly into existing clinical workflows; (3) digital solutions should function as shared coordination infrastructures that enable multistakeholder collaboration rather than as isolated documentation tools; and (4) digital tools should support task delegation, minimize additional workload, and enable bidirectional data exchange across care settings to ensure practical usability and sustained adoption. Based on these findings, a phased implementation strategy is recommended.

In the short term (0‐12 months), the SSED score should be embedded into existing primary care workflows by enabling MPAs to complete routine score components, thereby reducing GP workload. Crucially, this embedding should prioritize interoperability and data exchange over feature expansion. To mitigate fragmentation, the score should function as a shared digital coordination layer, enabling interoperable, bidirectional data exchange between GP systems, pharmacies, and patient-facing applications rather than operating as a standalone documentation tool.

In the medium term (12‐36 months), pharmacies could be integrated as decentralized hubs for standardized monitoring (eg, HbA_1c_ and cholesterol), a development supported by recent policy changes. In March 2025, the Swiss Parliament adopted a revision of the Health Insurance Act (Krankenversicherungsgesetz) enabling pharmacists to provide reimbursable services under mandatory health insurance from 2027 onward, including therapy adherence support, blood pressure screening, and medication optimization [42]. This policy shift aligns with evidence showing strong patient interest in pharmacy-based counseling, medication reviews, and treatment planning—services closely linked to quality indicators captured by the SSED score [36]. In parallel, individuals with T2D should be empowered as active contributors through self-tracking and tailored educational content linked to score results.

As a longer-term goal (>36 months), the SSED score should be expanded to reflect multimorbidity by incorporating additional cardiometabolic conditions commonly co-occurring with T2D, thereby increasing clinical relevance and sustainability in primary care. From a regulatory perspective, such a digital score would only fall under the Swiss Medical Device Regulation as a medical device if it provides automated decision support or treatment recommendations, whereas a descriptive, coordination tool would likely remain lower risk [43]. Future research should therefore evaluate clinical effectiveness, cost-effectiveness, professional adoption, and governance requirements as the score evolves toward multidisease and decision-support functionality.

Beyond provider-level changes, health insurers and public authorities are key enablers of adoption. Insurers can promote uptake by aligning reimbursement and incentives with coordinated care, for example, by rewarding SSED score use and supporting pharmacy-based monitoring pathways. Public authorities can facilitate scale-up by strengthening interoperability requirements. Together, these measures can help translate pilot models into routine practice.

More broadly, reimbursement structures will be critical for the sustainable implementation of digital quality monitoring. Payment models that primarily reward individual encounters may not adequately support data sharing, interprofessional coordination, and task delegation. Aligning incentives with coordinated, data-enabled care processes may therefore be essential for scaling digital quality monitoring.

Although this study focuses on Swiss primary care, the identified barriers—particularly fragmented data infrastructures, limited interoperability, and workflow misalignment—are common across many health care systems undergoing digital transformation [44]. The design principles derived from this study, such as prioritizing interoperable data exchange, integrating tools into existing clinical workflows, and supporting task redistribution, are therefore likely to be broadly transferable. However, the pace and feasibility of scale-up will depend on local conditions, including digital infrastructure maturity, regulatory frameworks, and reimbursement models [45,46]. Overall, while core design principles may generalize across settings, implementation strategies likely require adaptation to system-specific organizational and governance contexts.

### Theoretical Implications

Although this study followed an inductive qualitative approach, the findings can be interpreted through the lens of the technology acceptance model. The inductively derived themes map closely onto core technology acceptance model constructs, particularly perceived usefulness and perceived ease of use [15], while also extending them in ways that are highly relevant for digital health and chronic disease management. Perceived usefulness was shaped less by standalone technological features and more by workflow integration, interoperability with existing clinical systems, and the redistribution of tasks to MPAs or pharmacies. Likewise, perceived ease of use was closely linked to system-level integration and time savings within routine primary care workflows. Importantly, the findings indicate that technology acceptance in primary care is not solely an individual decision but a collective, ecosystem-level process influenced by interprofessional coordination, incentive structures, and patient engagement mechanisms. From a theoretical perspective, this suggests a need to extend the technology acceptance model in health care contexts to better account for organizational roles, reimbursement arrangements, and shared value creation across multiple stakeholders, thereby situating digital quality measurement tools within broader care models rather than treating them as isolated technologies.

### Limitations

This study has several limitations. First, although the sample included diverse professional backgrounds—such as health care professionals, individuals with T2D, health insurers, and health care software developers—the participants were not representatively sampled across Switzerland. Most interviewees came from the German-speaking region. This regional concentration limits the generalizability of findings, particularly related to system interoperability, IT integration, and data exchange practices, which may vary across regions due to differences in cantonal implementation and digital maturity in the region. Second, we acknowledge the possibility of participant self-selection, as those with a particular interest in digital health or structured diabetes care might have been more inclined to participate, thus introducing bias in the sample. Third, the study explored mostly hypothetical SSED score adoption scenarios. As such, many of the insights gathered are based on anticipated rather than observed behavior. This limits our ability to assess real-world effectiveness, feasibility, or unintended consequences. While participants reflected thoughtfully on enablers and barriers, their responses may not fully predict actual adoption behavior, especially under changing workload or policy conditions. While this study concentrated on identifying key challenges and generating initial solution ideas, future research should perform a thorough evaluation of how these essential features and adoption strategies could be designed and implemented.

Fourth, this study did not include community pharmacists as interview participants. While pharmacy-based follow-up emerged as a potentially important component of future diabetes care models in the ”Results” and “Discussion” sections, these suggestions originated from other stakeholder groups, including individuals with T2D, GPs, insurers, and digital health developers. As such, the findings reflect perceptions about the role of pharmacies rather than insights from pharmacists themselves. The absence of community pharmacists limits the extent to which conclusions can be drawn about the feasibility, acceptability, and operationalization of pharmacy-led follow-up, highlighting the need for future research to directly incorporate pharmacists’ perspectives.

Finally, economic considerations were discussed only at a conceptual level. Although stakeholders frequently raised cost concerns, particularly regarding time investment, IT integration, and the lack of direct reimbursement for MPAs without diabetes education, this study did not include quantitative modeling of the potential return on investment or long-term impact on health care usage. Further research using cost-effectiveness or budget impact analysis frameworks will be essential to substantiate the financial case for broader adoption.

### Conclusion

This study highlights the importance of aligning digital health innovations related to quality monitoring tools with the practical realities of clinical care. The SSED score, in a digital form, has the potential to enhance the quality and coordination of T2D management across Swiss primary care. However, our results suggest that successful adoption of a digital SSED score may depend on addressing key barriers identified by stakeholders: the need for system interoperability, time-saving features, delegation of tasks, and improved engagement. Our findings further suggest that technological sophistication and financial incentives for GPs alone are unlikely to be sufficient to drive change. Rather, the integration of an SSED score–based application must be supported by a multifaceted adoption strategy, combining workflow-compatible design, interprofessional collaboration, and task-shifting activities. New financing models that redistribute tasks to MPAs or pharmacies, empower individuals with T2D through self-monitoring, and enable real-time data sharing to offer a compelling path forward.

While these strategies are promising from a stakeholder perspective, their feasibility and effectiveness require validation in real-world settings. Future research should, therefore, move beyond hypothetical scenarios and empirically test digital SSED score–based applications through pilot implementations and accompanying economic evaluations to assess their impact on care processes, clinical and efficiency outcomes, and resource use.

## Supplementary material

10.2196/82960 Multimedia Appendix 1 Interview guides.

10.2196/82960 Multimedia Appendix 2 Interviewee’s background.

10.2196/82960 Checklist 1 COREQ checklist.

## Abbreviations

CIS: clinical information system
COREQ: Consolidated Criteria for Reporting Qualitative Research
EHR: electronic health record
GP: general practitioner
HbA_1c_: glycated hemoglobin
MPA: medical practice assistant
MPK: medical practice coordinator
RO: research objective
SSED: Swiss Society of Endocrinology and Diabetology
T2D: type 2 diabetes
TARDOC: Tarif für ambulante ärztliche Leistungen und Dokumentation
TARMED: tarif médical
