# Supplementary material for: Digital Quality Monitoring for Type 2 Diabetes in Swiss Primary Care: Qualitative Interview Study
Source: J Med Internet Res. 2026 May 12;28:e82960. doi: 10.2196/82960 (PMC13163630; doi:10.2196/82960)
Supplement: Multimedia Appendix 1 [file jmir-v28-e82960-s001.docx]

**Interview Guides**

**Individuals with T2D**

**Einführung Hintergrund**

- Wie alt sind Sie?
- Seit wie vielen Jahren haben Sie Diabetes?
- Haben Sie vor Ihrer Diagnose Hinweise oder Warnsignale für Typ-2-Diabetes wahrgenommen?
- Wurde Ihnen vor Ihrer Diagnose jemals empfohlen, präventive Maßnahmen zu ergreifen, wie z. B. Ernährungsumstellungen oder mehr Bewegung? Welche?
- Glauben Sie, dass Sie durch bestimmte Informationen oder Hilfestellungen früher hätten handeln können, um die Diagnose zu vermeiden? Wenn ja, welche?

**Status Quo: Allgemeine Diabetes-Kontrolle beim Hausarzt und Eigenverantwortung**

- Wie oft gehen Sie zum Hausarzt
- Wie würden Sie Ihre Beziehung zu Ihrem Hausarzt bzw. Ihrem Diabetes-Behandlungsteam beschreiben? Was funktioniert gut, und was könnte besser sein?
- Welche Faktoren beeinflussen, wie regelmäßig Sie Ihre Gesundheitswerte messen?
- wo erhalten Sie die meiste Unterstützung oder hilfreiche Informationen?“
- Wenn Sie nicht aktiv auf Ihre Gesundheitswerte (z.B. Blutdruck, LDL-C, BMI) achten, welche Hürden oder Herausforderungen hindern Sie daran?
- Wo speichern Sie Ihre Gesundheitsdaten ab? Würde Ihnen eine digitale Plattform helfen, den Überblick zu bewahren?
- Wenn Ihr Arzt Ihre Fortschritte mithilfe eines Scores verfolgen würde, würde das beeinflussen, wie Sie Ihren Diabetes managen?
- Gibt es Bereiche, in denen Sie zusätzliche von Ihrem Arzt Unterstützung benötigen?

**Unterstützung durch Technologien**

- Welche Rolle spielen digitale Tools und Gadgets in Ihrem Alltag? Besitzen sie bereits Gadgets wie Smartphone, Fitnessuhr oder Variable?
- Wenn Sie ein System oder eine Technologie verwenden könnten, um Ihr Diabetes-Management zu verbessern, welche Funktionen wären für Sie besonders wichtig und warum?
- Telemedizin: Könnten Sie sich vorstellen, dass wenn Sie gut eingestellt sind, gewisse Behandlungen telemedizinisch erledigen könnten?

**Lösgungsansätze**

- Gibt es bestimmte Erfahrungen oder Probleme, die Sie bisher noch nicht teilen konnten, die aber für Ihr Diabetes-Management relevant sind?
- Haben Sie Vorschläge, wie Hausärzte oder Gesundheitsdienstleister besser auf die Bedürfnisse von Menschen mit Typ-2-Diabetes eingehen könnten?
- Wie stellen Sie sich eine ideale Lösung vor, die Ihre individuellen Anforderungen im Diabetes-Management erfüllt?
- Welche Meinung haben Sie bezüglich Hilfsmitteln wie bspw. Support Gruppen oder Telemedizin?
- Was müsste das SGED Tool haben, damit dass Sie es benutzen würden?

**Health insurances**

**Einführung Hintergrund**

- Was arbeiten Sie und wie viele Jahre Berufserfahrung haben Sie?

**Übernahme von Kosten und Vergütungsmodellen**

- Welche Kosten für die Diabetesversorgung übernehmen Sie aktuell?
  - Wie werden Patienten die bereits T2D haben unterstützt?
  - Wie werden Patienten, die ein Risiko für T2D Risiko haben, präventiv unterstützt?
- Wie beurteilen Sie die potenziellen Kosteneinsparungen, wenn Hausärzte eine größere Rolle in der Diabetesversorgung übernehmen?

**Rolle und Motivation der Hausärzte in der Diabetesversorgung**

- Wie bewerten Sie die Rolle von Hausärzt*innen in der langfristigen Betreuung von Diabetespatient*innen?
- Welche finanziellen oder nicht-finanziellen Anreize könnten Hausärzte motivieren, sich stärker in der Diabetesversorgung zu engagieren?
- Welche Unterstützung könnten Krankenkassen in diesem Bereich bieten?

**Unterstützung von digitalen Lösungen**

- Welche Faktoren beeinflussen Ihre Entscheidung, ein digitales Gesundheits-Tool oder eine Plattform für die Diabetesversorgung zu unterstützen?
- Welchen Mehrwert sehen Sie in der Digitalisierung des SGED-Scores für die Diabetesversorgung?
- Welche weiteren Funktionen oder Tools (z. B. Versorgungspass, Anamnese-Dashboard) wären für Sie interessant, möglicherweise auch über Diabetes hinaus? (eine allgemeine Lösung für chronische Erkrankungen)

**Unterstützung und Vermarktung**

- Inwiefern sind Sie bereit, ein erfolgs- oder Outcome-basiertes Vergütungsmodell für Hausärzte zu unterstützen, welches die Qualität der Diabetesversorgung steigern könnte?
- Welche Maßnahmen könnten Ihrer Meinung nach die Akzeptanz solcher Systeme bei Hausärzten steigern?
- Würden Sie ein Bonus- oder Incentive-System für Hausärztinnen in Betracht ziehen, wenn Diabetespatientinnen nachweisbare Verbesserungen zeigen (z. B. bessere HbA1c-Werte oder weniger Komplikationen)?

**Weiterführende Themen**

- Gibt es zusätzliche Aspekte oder Bedenken, die Ihrer Meinung nach in der Diabetesversorgung stärker berücksichtigt werden sollten?
- Kennen Sie weitere wichtige Personen zu diesem Thema, mit welchen wir uns unbedingt austauschen sollten?

**Healthcare application developers**

**Einführung und Hintergrund**

- Seit wie vielen Jahren arbeiten Sie als Softwareproducer? (Jahre Berufserfahrung)
- Seit wie vielen Jahren arbeiten sie in der Gesundheitsbranche?

**Eigene bestehende Lösungen auf dem Markt**

- Bieten Sie bereits Lösungen für das Typ-2-Diabetes-Management an?

Ja:

- Wer ist Ihre Zielgruppe?
- Können Sie kurz erklären, wie Ihre Lösung funktioniert?
- Welche Vorteile bietet Ihre Lösung der Zielgruppe? Warum zeigt Ihre Zielgruppe Interesse an dieser Lösung?
- Welches Feedback erhalten Sie typischerweise von Ihrer Zielgruppe? Gibt es konkrete Wünsche oder Kritikpunkte, die Ihnen dabei helfen, Ihre Lösung zu verbessern?
- Wie positionieren Sie Ihre Lösung im Vergleich zu anderen Anbietern auf dem Markt?
- Was kostet Ihre Lösung?
- Unterstützt Ihre Lösung die Förderung des SGED-Scores? Falls ja, wie wird dies unterstützt?

Nein:

- Haben Sie schon einmal über die Entwicklung einer Diabetes-Management-Lösung nachgedacht? Wenn ja, warum wurde dies nicht weiterverfolgt?
- Wie bewerten Sie den Markt für Typ-2-Diabetes-Management-Lösungen? Gibt es Ihrer Meinung nach ausreichend Bedarf oder Lücken, die eine neue Lösung füllen könnte?
- Sehen Sie potenzielle Hindernisse oder Herausforderungen bei der Entwicklung oder Einführung einer solchen Lösung?
- Welche Faktoren halten Sie bisher davon ab, in diesem Bereich aktiv zu werden (z. B. Ressourcen, Prioritäten, Marktunsicherheit)?
- Würden Ihre bestehenden Produkte oder Technologien es erleichtern, eine Lösung für das Typ-2-Diabetes-Management zu entwickeln? Falls ja, wie?

**Einbindung in bestehende Applikationen durch Schnittstellen**

- Können Ihre Lösungen durch Schnittstellen (APIs) erweitert oder angepasst werden?
- Was wären die Hauptgründe, aus denen Sie unsere Lösung oder eine eigene Lösung in Ihre bestehenden Applikationen integrieren würden?
- Welche Herausforderungen sehen Sie bei der Integration einer neuen Lösung in Ihre bestehenden Systeme?
- Welche besonderen Anforderungen hinsichtlich Datenschutzes und Sicherheit müssen bei der Integration von Lösungen im Gesundheitsbereich berücksichtigt werden?
- Welche Rolle spielt für Sie die Fähigkeit einer Software, mit anderen Gesundheitslösungen (z. B. Patientenakten) nahtlos zu interagieren?

**Potenzial für zukünftige Lösungen**

- Gibt es bestimmte Features oder Technologien, die Sie in einer idealen Diabetes-Management-Lösung für relevant halten würden?
- Welche Rolle könnten innovative Technologien wie Künstliche Intelligenz, Machine Learning oder IoT in diesem Bereich spielen?
- Wie sehen Sie die Entwicklung von Diabetes-Management-Software in den kommenden Jahren? Welche Trends und Technologien halten Sie für besonders vielversprechend?
- Welche weiteren Features oder Tools würden Sie gerne in einer idealen Typ-2-Diabetes-Management-Lösung sehen?
- Haben Sie weitere Ideen oder Bedenken, die wir bei der Entwicklung oder Erweiterung einer Diabetes-Management-Lösung berücksichtigen sollten?
- Kennen Sie weitere wichtige Personen zu diesem Thema, mit welchen wir uns unbedingt austauschen sollten?

**Healthcare professionals (GPs and MPAs)**

**Einführung und aktueller Status quo**

- Seit wie vielen Jahren arbeiten Sie als Hausarzt/Hausärztin (Jahre Berufserfahrung)
- Wie viel Prozent Ihrer Patienten haben Diabetes (Typ 2)? Hätten Sie gerne mehr oder weniger? Wie aufwendig sind für Sie diese Patienten? Wie lukrativ sind Diabetes-Patienten für Sie?
- Können Sie kurz beschreiben, wie Sie derzeit Ihre Diabetespatienten betreuen? Sehen Sie hier eine besondere Verantwortung oder Herausforderung im Vergleich zu anderen Patientengruppen?
- Nutzen Sie (oder Ihre Patienten) aktuell digitale Tools für das Diabetesmanagement? Falls ja, welche?
- Verwenden Sie den SGED-Score in Ihrer Praxis? Warum, warum nicht? Wenn ja, wie setzen Sie ihn ein?
- Wie „sattelfest“ fühlen Sie sich in der Betreuung der Patienten? Wie oft pro Jahr gehen Sie an Weiterbildungen zu dem Thema? (Zahlen Sie diese selbst?) Haben Sie regelmässig Austausch mit Diabetologen zu dem Thema?

**Wahrgenommener Nutzen und Benutzerfreundlichkeit**

- Welche (potentiellen) Vorteile sehen Sie im Einsatz digitaler Tools für die Betreuung von Diabetespatienten? Gibt es bestimmte Informationen oder Funktionen, die Ihnen ein digitales Tool zur Unterstützung Ihrer Arbeit bieten sollte?
- Falls SGED verwendet wird: Wie könnte eine digitale Version des SGED-Scores Ihre Arbeit effizienter gestalten?
  - Gäbe es eine Möglichkeit, dass MPAs die Daten zuerst aufnehmen?
- Welche Herausforderungen sehen Sie bei der Nutzung digitaler Tools im Diabetesmanagement? Haben Sie bereits auch frustrierende Erfahrungen gemacht (mit digitalen Tools allgemein)?
- Was denken Sie macht ein Tool besonders einfach und praktikabel? *Und* was erschwert die Nutzung digitaler Tools in Ihrer täglichen Arbeit? (Auch bezüglich deren Implementierung)
- Wie viel Zeit würden Sie in das Erlernen eines neuen Tools investieren?

### **Barrieren, Lösungen, Motivation und Anreize zur Nutzung**

- Was hindert Sie oder Ihre Kollegen daran, digitale Tools intensiver zu nutzen?
- Was würde Sie motivieren, ein neues Tool auszuprobieren und in Ihrer Praxis anzuwenden?
- Wenn Sie auswählen könnten: Welcher der folgenden Anreize würde Sie am meisten motivieren ein solches Tool zu adoptieren:
  - eine **erfolgsbasierte Vergütung,** bei der Sie für messbare Verbesserungen in den Diabetes-Parametern Ihrer Patient*innen belohnt werden (von Krankenkasse, zB via managed Care Netzwerke)
  - Auszeichnung von leistungsstarken Praxen als „**Centers of Excellence“;** Krankenkassen würden diese an Patienten empfehlen.
  - Die Möglichkeit, anonymisierte Patientendaten mit anderen **Hausarztpraxen** zu teilen und sich gegenseitig durch Coachings zu unterstützen.
  - Die Möglichkeit, anonymisierte Patientendaten mit anderen **Diabetologen** zu teilen und von ihnen Coachings zu erhalten.
  - **Optimierte Prozesse** im Diabetes-Management Ihrer Patient*innen, die Ihnen wertvolle Zeit einsparen.
- Hätten Sie noch weitere Ideen, was Sie motivieren, würde für die Nutzung dieses Tools?

### **Empfehlungen und Zukunftsperspektiven**

- Welche Empfehlungen hätten Sie für die Entwickler digitaler Tools, damit diese für Hausärzte attraktiver und nützlicher werden?
- Wie sehen Sie die Rolle digitaler Tools in der Zukunft der Diabetesversorgung?

### **Abschluss**

- Welche Patienteninformationen sind wichtig, um das Diabetesmanagement voranzutreiben?
  - Sind ihre Patienten technologieaffin? (Patienteninformationen müssen im Alltag durch Smartphones, Fitness band oder variables gesammelt werden)
  - Würden Patienten solche Technologien verwenden?

**Healthcare professionals (Endocrinologist)**

1. **Einstieg / Einführung (3–5 Minuten)**

- Vielen Dank, dass Sie sich Zeit für dieses Gespräch nehmen.
- Ich führe derzeit Interviews im Rahmen einer Studie durch, in der es um die bessere Integration des SGED-Scores in der Schweizer Grundversorgung geht. Besonders interessiert mich Ihre Sicht als Endokrinolog:in und Ihre Erfahrungen mit der strukturierten Diabetesversorgung.
- Ihre Einschätzungen helfen uns dabei, besser zu verstehen, warum der SGED-Score bisher wenig genutzt wird – und was sich ändern müsste, damit er häufiger eingesetzt wird.
- Das Gespräch dauert rund 30 Minuten. Alles, was Sie sagen, wird vertraulich behandelt und anonymisiert.
- Wäre es für Sie in Ordnung, wenn ich das Gespräch zu Transkriptionszwecken aufzeichne?

1. **Teil 1: Erfahrung mit dem SGED-Score und Zusammenarbeit mit Hausärzt:innen (5–7 Minuten)**

- Was ist aus Ihrer Sicht der ursprüngliche Zweck oder Mehrwert des SGED-Scores?
- Wie erleben Sie die aktuelle Versorgung von Typ-2-Diabetes durch Hausärzt:innen?
- Wie häufig und auf welche Weise arbeiten Sie mit Hausärzt:innen bei Diabetespatient:innen zusammen?
- Weshalb, glauben Sie, wird der SGED-Score bisher kaum in der Grundversorgung angewendet?
- Was hören Sie von Hausärzt:innen in Bezug auf den Score – wenn überhaupt?
- Gibt es systemische oder organisatorische Hürden, die eine Umsetzung erschweren?
- Beobachten Sie eine Diskrepanz zwischen den Empfehlungen von Spezialist:innen und dem, was Hausärzt:innen im Alltag umsetzen können?

1. **Teil 2: Anreizsysteme und Motivation (5–7 Minuten)**

- Welche Anreizsysteme (finanzieller oder nicht-finanzieller Art) könnten Ihrer Meinung nach Hausärzt:innen motivieren, den SGED-Score stärker zu nutzen?
- Glauben Sie, dass Qualitätsmessung wie der SGED-Score mit Vergütung oder Boni verknüpft werden sollte? Warum bzw. warum nicht?
- Könnten auch nicht-monetäre Anreize wie Benchmarking, fachliche Unterstützung oder digitale Tools eine Rolle spielen?

1. **Lösungsansätze und Empfehlungen (5–6 Minuten)**

- Was müsste sich Ihrer Meinung nach ändern, damit der SGED-Score in der Hausarztpraxis häufiger eingesetzt wird?
- Könnte es eine Möglichkeit sein, Patienten und andere Stakeholder (e.g. Apotheken) mehr zu involvieren, damit Hausärzte nicht so stark unter Wasser sind mit dem Sammeln der Daten?
- Gibt es aus Ihrer Sicht gute Praxisbeispiele – aus dem In- oder Ausland –, von denen wir lernen könnten?

1. **Abschluss (1–2 Minuten)**

- Gibt es sonst noch etwas, das Sie zum Thema SGED-Score oder zur strukturierten Diabetesversorgung in der Schweiz anmerken möchten?
