# Supplementary material for: Digital Quality Monitoring for Type 2 Diabetes in Swiss Primary Care: Qualitative Interview Study
Source: J Med Internet Res. 2026 May 12;28:e82960. doi: 10.2196/82960 (PMC13163630; doi:10.2196/82960)
Supplement: Multimedia Appendix 2 [file jmir-v28-e82960-s002.docx]

**Table 1.** Overview of the interviews with the healthcare professionals.

| Participant | Duration of Interview | Years of Experience |
| --- | --- | --- |
| GP 1 | 46 min | 22 |
| GP 2 | 39 min | 30 |
| GP 3 | 31 min | 24 |
| GP 4 | 35 min | 26 |
| GP 5 | 34 min | 25 |
| GP 6 | 65 min | 31 |
| GP 7 | 37 min | 31 |
| GP 8 | 28 min | 0.5 |
| GP 9 | 29 min | 10 |
| GP 10 | 37 min | 27 |
| PT1 | 65 min | 24 |
| Endocrinologist | 21 min | 25 |

**Table 2.** Overview of the interviews with T2D individuals.

| Participant | Duration of interview | Age |
| --- | --- | --- |
| P1 | 30 min | 56 |
| P2 | 38 min | 70 |
| P3 | 31 min | 73 |
| P4 | 29 min | 51 |
| P5 | 28 min | 65 |
| P6 | 31 min | 74 |
| P7 | 22 min | 75 |
| P8 | 30 min | 40 |
| P9 | 29 min | 63 |
| P10 | 35 min | 70 |
| P11 | 31 min | 68 |
| P12 | 29 min | 58 |

**Table 3.** Overview of the interviews with healthcare software developers

| Participant | Duration of Interview | Interviewee’s position |
| --- | --- | --- |
| SP1 | 49 min | Managed Care |
| SP2 |  | Product Manager |
| SP3 | 41 min | Chief Medical Officer |
| SP4 | 36 min | Chief Executive Officer |
| SP5 | 28 min | Chief Operating Officer |
| SP6 | 55 min | Senior Consultant |
| SP7 | 48 min | International Training & Education Leader |
| SP8 | 27 min | Chief Executive Officer |
| SP9 | 32 min | Chief Executive Officer |
| SP10 | ~ 30 min | Responsible Chronic Care Management, Organisation of education |

**Table 4.** Overview of the interviews with the health insurer companies

| Participant | Duration of Interview | Interviewee’s position |
| --- | --- | --- |
| HI1 | 37 min | Strategie & Corporate Services |
| HI2 | 39 min | Product manager OKP |
| HI3 | 31 min | Head of Sourcing & Procurement |
| HI4 | 31 min | Lead integrated care |
| HI5 | 25 min | Head of Medical Services Procurement |
